# Dabrafenib Plus Trametinib: An Impressive Response in an Adult Patient With BRAF V600E-Mutated and Isocitrate Dehydrogenase (IDH) Wild-Type Glioma

**DOI:** 10.7759/cureus.28156

**Published:** 2022-08-19

**Authors:** Inês N Costa, Joana Reis, Jorge Pinheiro, Roberto Silva, Catarina Fernandes

**Affiliations:** 1 Medical Oncology, Centro Hospitalar Universitário de São João, Porto, PRT; 2 Pathology, Centro Hospitalar Universitário de São João, Porto, PRT

**Keywords:** glioma, idh wildtype, astrocytoma with piloid features, cdkn2a/b, dabrafenib plus trametinib, braf v600e

## Abstract

Key molecular alterations found in the diagnosis and prognosis of brain tumours have been revealed by the latest advances in transcriptomic and genome-wide analysis. In-depth studies revealed that alterations of the V-Raf murine sarcoma viral oncogene homolog B (BRAF) could be shared by different brain tumour types. The identification of BRAF p.V600E mutations in gliomas is nowadays of more importance regarding the development of BRAF-targeted inhibitors.

This report presents the case of a 37-year-old female with a voluminous expansive neoplastic lesion, extending from the lenticulocapsular region to the medial aspect of the temporal lobe on the left. Pathological examination revealed an astrocytic neoplasm without high-grade histological features in small biopsy fragments. The molecular study revealed the presence of a mutation in the BRAF V600E gene and CDKN2A/2B homozygous deletion. The lesion was partially removed and irradiated. The patient has been on treatment with dabrafenib plus trametinib for 10 months. In addition to reasonable tolerance, she obtained an impressive tumour reduction, which was manifested in the complete resolution of neurological deficits and in the full acquisition of autonomy.

The remarkable results reported in this clinical case justify the pressing need to identify new therapeutic targets in gliomas in the current era of precision medicine.

## Introduction

Gliomas and glioneuronal tumours are the most prevalent group of intrinsic central nervous system (CNS) neoplasms. This category of tumours comprises a huge diversity of pathological types with diverse prognoses. Moreover, molecular studies, namely wide genome sequencing and methylation profiling, have been updating the knowledge about these CNS tumours and the recent revised fifth edition of the World Health Organization (WHO) classification included new, well-established molecular parameters in the glioma classification. Some specific molecular characteristics have been introduced in the definition of tumour types (i.e. IDH1/2 and histone H3 mutations) and others in grading criteria (i.e. CDKN2A/2B homozygous deletion) [1].

This new integrative approach to the classification and grading of gliomas significantly improved the prediction of prognosis. In specific cases, it is also dependent on patient factors, such as the age of the patient and the location and size of the tumour. However, the therapeutic options for gliomas treatment remain limited [2], and only a few target therapies are available.

V-Raf murine sarcoma viral oncogene homolog B1 (BRAF) gene alterations may be held in primary brain tumours [3]. The BRAF gene, located at chromosome 7 (7q34), is a main mediator in the mitogen-activated protein kinase (MAPK) cell signalling pathway and encodes the B-Raf protein, a serine/threonine protein kinase working downstream of the Ras-Raf-MEK-ERK signalling pathway; it is an intracellular signal transducer between extracellular growth stimuli and cellular response [3]. p.V600E is a highly reported oncogenic mutation driver in BRAF (~90%) and occurs through a point mutation (p.T1799A) in exon 15 of BRAF, ending up in the substitution of valine with glutamic acid at codon 600 in most cases [4]. V600E, encountered inside the activation region of BRAF, leads to constitutive activation of the MAPK signalling pathway, leading to hyperactivation (~500x) of the signalling cascade and the uncontrolled division of cells, from insensitivity to negative feedback mechanisms and consequent tumorigenesis [4].

The BRAF p.V600E mutation is not specific to a tumour type. It is commonly held in the category of pleomorphic xanthoastrocytoma and ganglioglioma and can also be found in a subset of pilocytic astrocytomas (PA) and glioblastomas (namely with epithelioid morphology) [3]. Adult patients have shown been to have a lower prevalence of the BRAF p.V600E mutation when compared to paediatric patients (4 vs 7%) [4].

BRAF inhibitors, which are small kinase inhibitors, bind specifically to V600E-mutated B-Raf proteins and do not allow them to activate MEK, consequently stopping the MAPK/ERK signalling cascade, inhibiting aberrant cell signalling. MEK inhibitors applied during therapy delay developing resistance to BRAF inhibitors and increase the time of tumour progression [4]. So far, data on the effectiveness of this targeted treatment in gliomas come from phase 2 trials, systematic review, and clinical case descriptions.

We present an adult-onset case of BRAF V600E-mutated extensive astrocytoma, conditioning a much higher burden of symptoms, partially resected and treated with a combination of BRAF and MEK inhibitors (BRAF+MEKi).

## Case presentation

A 37-year-old female with a history of papillary thyroid carcinoma operated in 2014 and currently in surveillance, was admitted to the emergency department for occipital headache and vomiting. A spontaneous subarachnoid haemorrhage was diagnosed.

**Figure 1:**
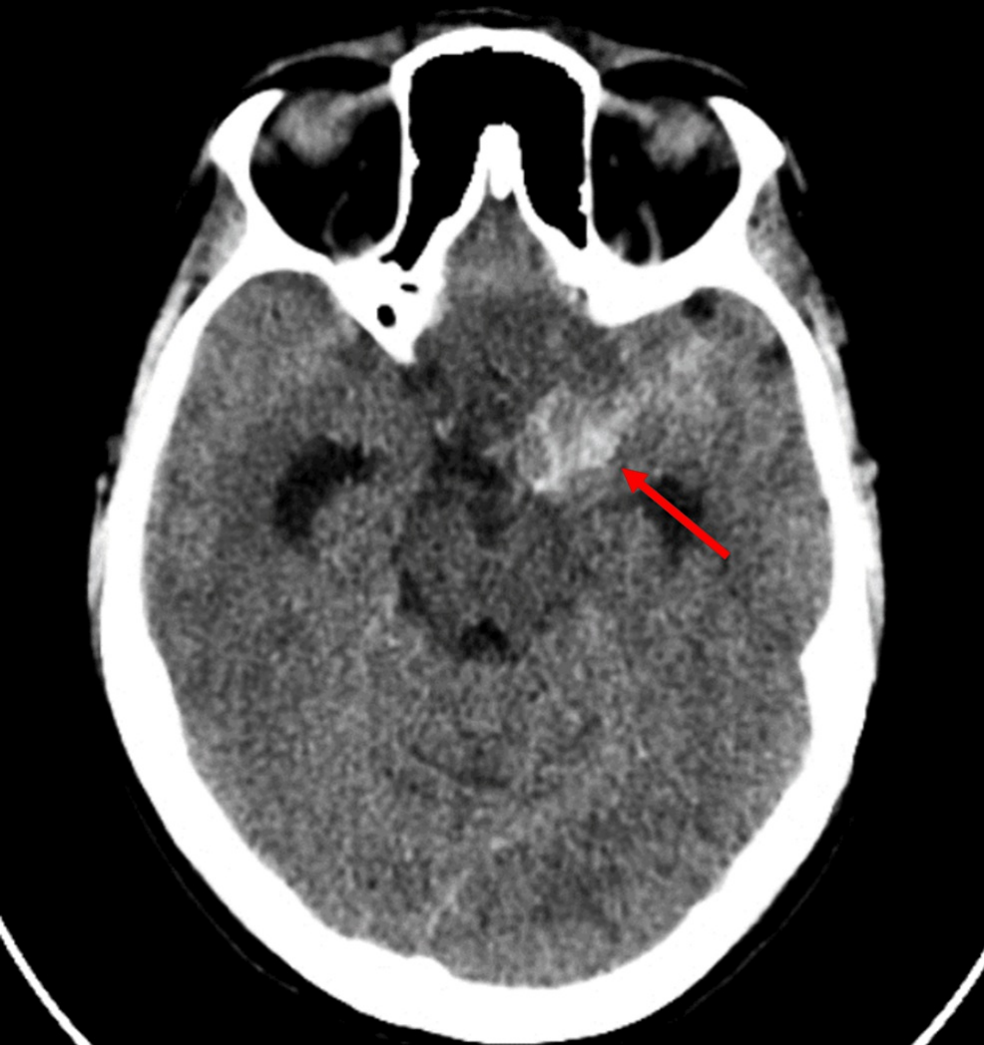
Brain CT Diffuse subarachnoid haemorrhage (arrow) in the basal cistern, predominantly on the left side of the pentagonal cistern

Brain CT showed a diffuse subarachnoid haemorrhage in the basal cistern, predominantly on the left side of the pentagonal cistern and with an important hematic component in the left Sylvian trench and grooves of the hemilateral temporo-frontal convexity (Figure 1). Angiography showed no pathological changes, namely cerebral aneurysms, signs of vasospasm, or images suggestive of arteriovenous shunts. No neurological deficits and almost-complete reabsorption of the haemorrhage were observed at the time of discharge.

**Figure 2:**
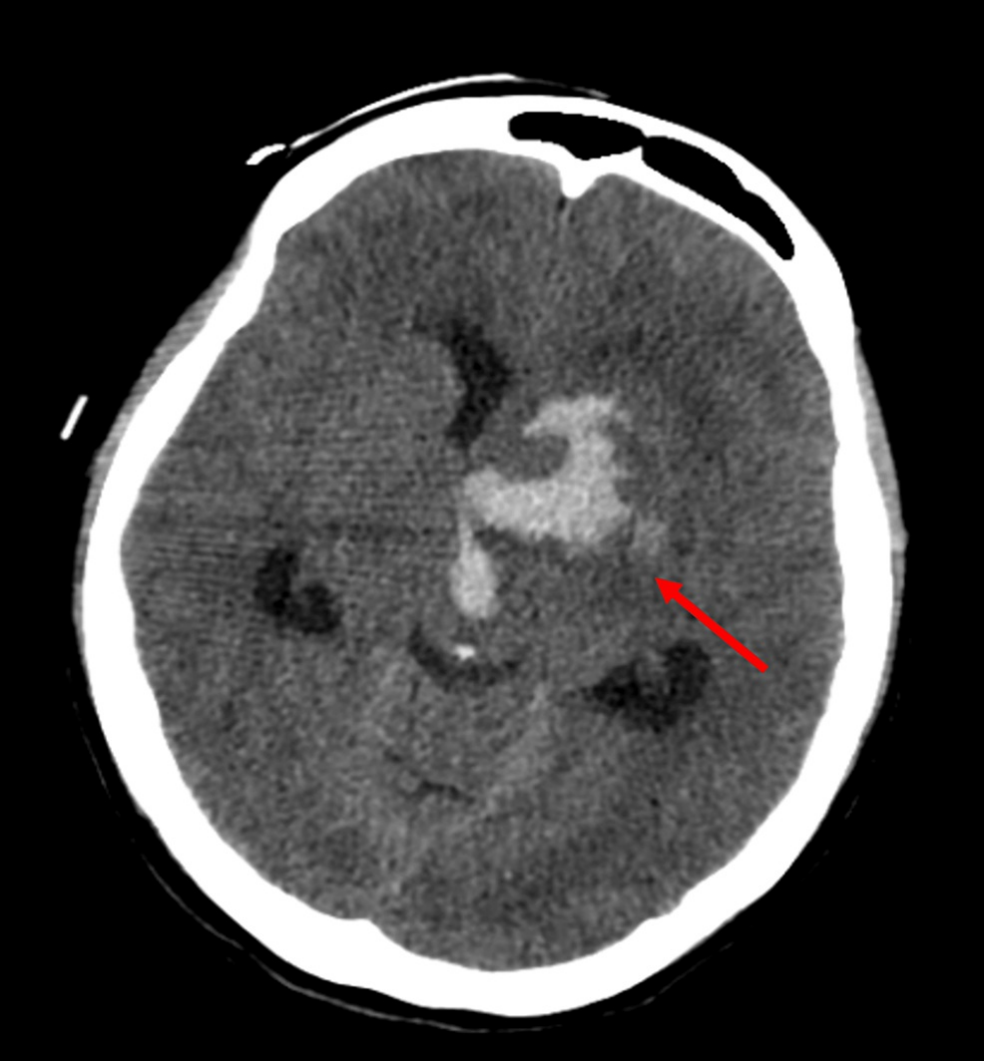
Brain CT Haemorrhagic lesion (arrow) in the left lenticulocapsular region and tetraventricular haemorrhage with ectasia of the left ventricular system and moderate hydrocephalus

After three months, the patient was admitted to the emergency room due to loss of consciousness and right hemiplegia. Brain CT showed an acute-phase haemorrhagic lesion with an epicentre in the left lenticulocapsular region, where a hematoma, about 25 mm in diameter, was observed with dissection into the ventricular system, showing tetraventricular haemorrhage. Left uncal hernia and left lateral mesencephalic obliteration were seen. Ectasia of the left ventricular system with moderate hydrocephalus was visualised (Figure 2). An external ventricular drain was placed.

**Figure 3:**
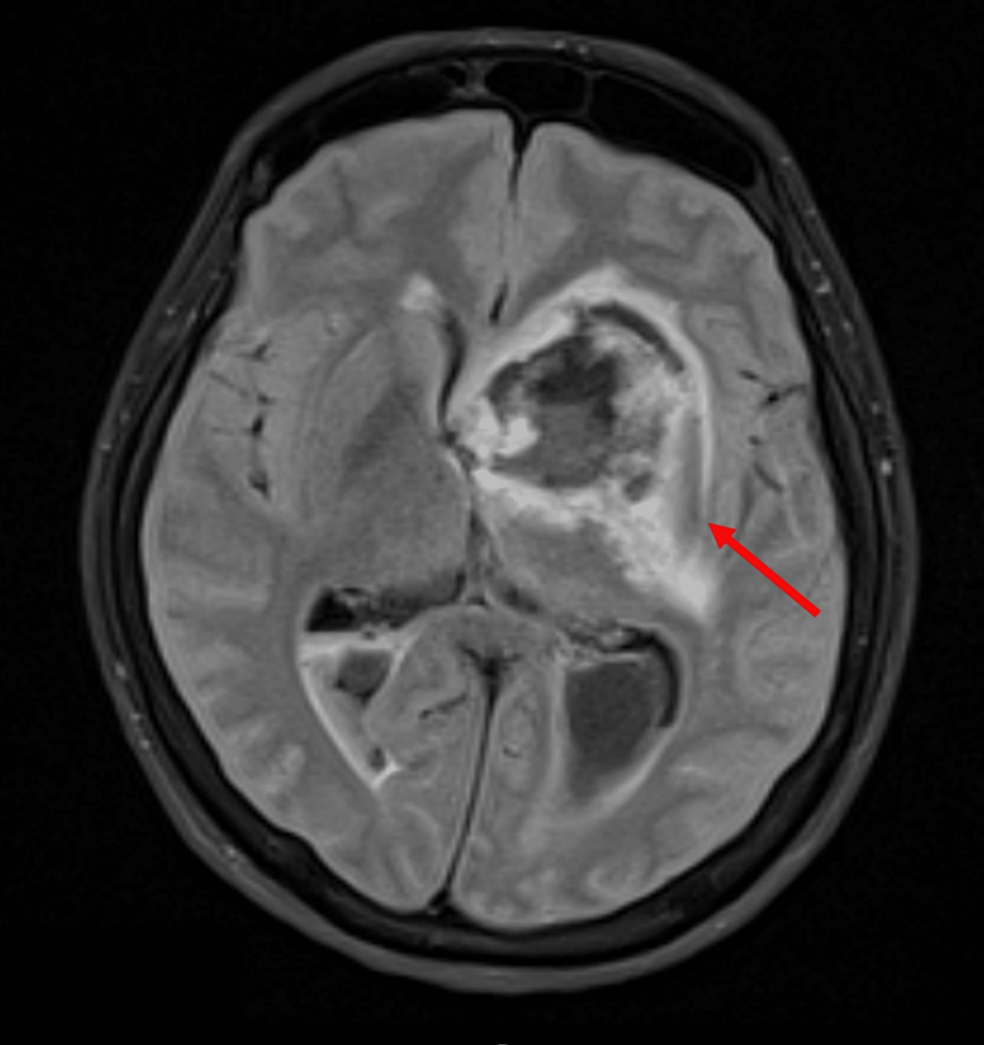
Brain MRI A neoplastic lesion (arrow), extending from the lenticulocapsular region to the medial aspect of the temporal lobe on the left, presenting perilesional oedema and a mass effect on the third ventricle and the left lateral ventricle, with a deviation of the median structures to the right

Brain MRI showed an expansive neoplastic lesion, extending from the lenticulocapsular region to the medial aspect of the temporal lobe on the left, with a rounded configuration, measuring 43 x 40.3 x 29 mm. It had an opening superior cavity where it was related to a voluminous left striatocapsular intraparenchymal hematoma (31.7 x 35.3 x 36 mm). It presented perilesional oedema and a clear mass effect on the third ventricle and the left lateral ventricle with a deviation of the median structures to the right of about 5.5 mm (Figure 3).

**Figure 4:**
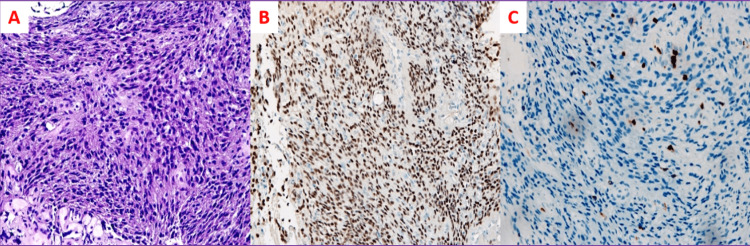
Histological Report A (haematoxylin and eosin (H&E), 200x) - Histological examination revealed very small fragments of a cellular glial tumour, composed of astrocytic cells with oval to elongated nuclei, with vesicular chromatin; B - Diffuse Olig2 expression (200x); C - Ki67 index was up to 10%

She underwent trans-Sylvian subfrontal access for biopsy and partial lesion removal. The histological result revealed very small fragments of a glial neoplasm with an astrocytic phenotype, of moderate to high cellularity. Neoplastic cells disclosed oval to elongated nuclei with vesicular chromatin and fibrillary cytoplasm. No ganglion cells or pleomorphic lipidized cells were identified. No mitosis was observed in the small product of the biopsy or areas of necrosis or microvascular proliferation. In the immunohistochemical study, diffuse olig2 and weak and focal expression of glial fibrillary acidic protein (GFAP) were observed in neoplastic cells. No expression of H3 p.K27M or IDH-1 p.R132H was identified; ATRX expression was preserved; p53 expression was wildtype. The proliferative index assessed by the ki-67 index was up to 10% (Figures 4A, 4B, 4C).

The next-generation sequencing (NGS) of the tumour revealed a mutation in the BRAF Val600Glu (V600E) gene. Homozygous deletion of CDKN2A/B genes was also detected. No evidence of alternative IDH1 or IDH2 mutations, EGFR amplification, or TERT promoter mutation was identified.

The case was presented at the multidisciplinary meeting. Given the aggressiveness of the lesion and the impossibility of complete surgical resection, radiotherapy was proposed.

She underwent external radiotherapy with a total dose of 54Gy, at 2Gy/day, in 27 fractions, with photon energy of 6MV, according to the three-dimensional conformal radiation therapy (3D CRT) technique.

Considering the context of glioma with the characteristics of aggressiveness and chemoresistance and the molecular profile of homozygous deletion of the CDKN2A/B genes and BRAF gene mutation (Val600Glu), she was proposed for treatment with dabrafenib plus trametinib. Before starting treatment, the patient had dysphasia, hypesthesia and right hemiparesis grade 4. She required Prednisolone at a dose of 30 mg, daily, orally, for better symptomatic control.

Dabrafenib 150 mg, twice daily, plus trametinib 2 mg, once daily, orally was started. She presented optimal tolerance and adherence, without adverse effects. She gradually reduced corticosteroid therapy until discontinuation.

**Figure 5:**
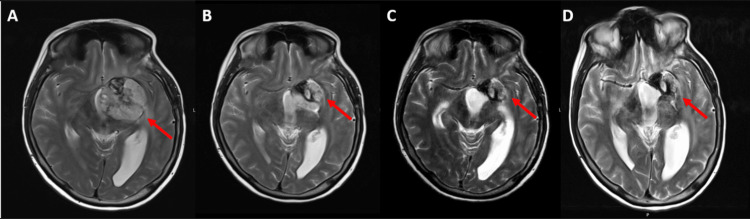
Sequential brain MRI The lesion-reducing effect of dabrafenib plus trametinib (arrows) A - Before the beginning of treatment; B - Three months after treatment; C - Six months after treatment; D - Nine months after treatment

The MRI reassessment performed five months after treatment was started, showed a decrease in the dimensions of the neoplastic lesion (26 x 20 mm larger in diameters). The last MRI showed stable disease. The imaging evolution from the beginning of the treatment to the last MRI can be seen in Figures 5A, 5B, 5C, 5D.

Currently, the patient is autonomous in daily life activities, with no deficit in strength or language disorders.

## Discussion

Brain tumours are more responsible for shortening years of life as compared to any other cancer [2]. Such calls for novel approaches in precision medicine have been detailed by biomarker studies. The histological classification of brain tumours will now incorporate molecular information with prognostic, predictive, and therapeutic values [1].

Although the current available molecular studies do not allow a final classification, the histological features and the co-occurrence of a BRAF p.V600E mutation and CDKN2A/2B homozygous deletion raises the possibility of a high-grade astrocytoma with piloid features (HGAPF) [5]. Methylation profiling would be necessary for a final diagnosis.

The diagnosis of the newly recognized HGAPF is challenging due to the non-specific histological and molecular features which they share with PA MAPK pathway gene alterations. Moreover, they may present anaplastic histological features, ATRX mutation or loss of expression (up to 40% of cases), homozygous deletion of CDKN2A/B (up to 80% of cases), and aneuploidy, factors that were previously recognized as aggressive behaviour markers in PA in previous studies [6].

PA therapy for adults lies on resection, although chemotherapy, radiation, or bevacizumab may also be considered [6]. Full resection may be curative. Although PA is considered a grade 1 neoplasm, it may cause life-threatening symptoms such as brainstem compression and hydrocephalus. Recurrence and progression of symptoms are possible if resection is incomplete [7].

On the other hand, the prognosis in HGAPF is only available in one retrospective study, with a five-year survival rate of approximately 50% [5]. More data are required for the assignment of a definitive CNS WHO grade, but current data suggest a clinical behaviour roughly corresponding to CNS WHO grade 3. Consensus on the proper therapeutic approach to this new tumour type remains to be defined.

The effectiveness of the BRAF and MEK inhibitors combination in BRAF V600E mutated gliomas treatment is shown in the phase 2 studies and reported in a systematic review [4, 8-9]. Preliminary results from one of these ongoing studies reported an objective response in 69% of patients with low-grade glioma who received dabrafenib 150 mg, twice daily, plus trametinib 2 mg, once daily, orally, until unacceptable toxicity, disease progression, or death [8].

The presence of CDKN2A/B deletion is associated with worse prognosis and inferior response to chemotherapy, regardless of the co-existence of mutations in the BRAF or IDH1/2 genes [9]. However, it is important to note that in patients with gliomas positive for the BRAF mutation, the presence of homozygous deletion of CDKN2A/B genes does not compromise the response to BRAF inhibitors [10].

One question that may be asked is what exact role radiotherapy played in response achievement. It certainly influenced tumour reduction, but as no imaging reassessment was performed before starting treatment with BRAF+MEKi, it does not allow us to confirm its exact contribution.

This case exemplifies that dabrafenib plus trametinib allow achieving an objective response and significant clinical improvement, with an acceptable toxicity profile which is quite surprising given the considerable size of the lesion and the initial symptom burden. For now, efficacy data from studies using BRAF+MEKi seem to be promising, but phase three trials are lacking to give more robustness to its use. Furthermore, there are still a few case reports in the literature sharing experience with this therapy, so the remarkable results described in our study make it unique.

Further studies are needed to clarify subsequent treatment in the case of progression and to recognize the mechanisms of resistance that may arise.

## Conclusions

This case reflects the importance of performing molecular screening as a means to identify new therapeutic targets. Only in this way was it possible for us to detect the presence of the BRAF p.V600E mutation and use targeted treatment. Our study portrays the effectiveness of BRAF+MEKi in an adult patient with a BRAF V600E mutation-positive glial neoplasm.
